# Treatment with apolipoprotein A-1 mimetic peptide reduces lupus-like manifestations in a murine lupus model of accelerated atherosclerosis

**DOI:** 10.1186/ar3020

**Published:** 2010-05-18

**Authors:** Jennifer MP Woo, Zhuofeng Lin, Mohamad Navab, Casey Van Dyck, Yvette Trejo-Lopez, Krystal MT Woo, Hongyun Li, Lawrence W Castellani, Xuping Wang, Noriko Iikuni, Ornella J Rullo, Hui Wu, Antonio La Cava, Alan M Fogelman, Aldons J Lusis, Betty P Tsao

**Affiliations:** 1 Department of Medicine-Rheumatology, David Geffen School of Medicine, University of California, 1000 Veteran Avenue, Los Angeles, CA 90095, USA; 2 Department of Medicine-Cardiology, David Geffen School of Medicine, University of California, 1000 Veteran Avenue, Los Angeles, CA 90095, USA; 3 Department of Medicine, Department of Microbiology, Immunology, and Molecular Genetics, Department of Human Genetics, David Geffen School of Medicine, University of California, 1000 Veteran Avenue, Los Angeles, CA 90095, USA; 4 Department of Pediatrics-Rheumatology, David Geffen School of Medicine, University of California, 1000 Veteran Avenue, Los Angeles, CA 90095, USA; 5 Department of Medicine, David Geffen School of Medicine, University of California, 1000 Veteran Avenue, Los Angeles, CA 90095, USA

## Abstract

**Introduction:**

The purpose of this study was to evaluate the effects of L-4F, an apolipoprotein A-1 mimetic peptide, alone or with pravastatin, in apoE^-/-^Fas^-/-^C57BL/6 mice that spontaneously develop immunoglobulin G (IgG) autoantibodies, glomerulonephritis, osteopenia, and atherosclerotic lesions on a normal chow diet.

**Methods:**

Female mice, starting at eight to nine weeks of age, were treated for 27 weeks with 1) pravastatin, 2) L-4F, 3) L-4F plus pravastatin, or 4) vehicle control, followed by disease phenotype assessment.

**Results:**

In preliminary studies, dysfunctional, proinflammatory high-density lipoproteins (piHDL) were decreased six hours after a single L-4F, but not scrambled L-4F, injection in eight- to nine-week old mice. After 35 weeks, L-4F-treated mice, in the absence/presence of pravastatin, had significantly smaller lymph nodes and glomerular tufts (*P*_*L*, *LP *_< 0.05), lower serum levels of IgG antibodies to double stranded DNA (dsDNA) (*P*_*L *_< 0.05) and oxidized phospholipids (oxPLs) (*P*_*L*, *LP *_< 0.005), and elevated total and vertebral bone mineral density (*P*_*L*, *LP *_< 0.01) compared to vehicle controls. Although all treatment groups presented larger aortic root lesions compared to vehicle controls, enlarged atheromas in combination treatment mice had significantly less infiltrated CD68^+ ^macrophages (*P*_*LP *_< 0.01), significantly increased mean α-actin stained area (*P*_*LP *_< 0.05), and significantly lower levels of circulating markers for atherosclerosis progression, CCL19 (*P*_*L*, *LP *_< 0.0005) and VCAM-1 (*P*_*L *_< 0.0002).

**Conclusions:**

L-4F treatment, alone or with pravastatin, significantly reduced IgG anti-dsDNA and IgG anti-oxPLs, proteinuria, glomerulonephritis, and osteopenia in a murine lupus model of accelerated atherosclerosis. Despite enlarged aortic lesions, increased smooth muscle content, decreased macrophage infiltration, and decreased pro-atherogenic chemokines in L-4F plus pravastatin treated mice suggest protective mechanisms not only on lupus-like disease, but also on potential plaque remodeling in a murine model of systemic lupus erythematosus (SLE) and accelerated atherosclerosis.

## Introduction

Premenopausal women with systemic lupus erythematosus (SLE or lupus) are at an estimated 10- to 50-fold increased risk for developing myocardial infarction and cardiovascular disease (CVD) compared to age-matched controls [1-3]. Moreover, subclinical atherosclerosis is more prevalent in women with SLE, as measured by carotid plaques [4] and coronary artery calcification [5,6]. Traditional Framingham risk factors for atherosclerosis cannot fully account for accelerated atherosclerosis in SLE [1], which is also influenced by SLE-related factors [7-9]. These SLE-related factors, including the use of corticosteroid therapy, chronic inflammation, and the extent of disease damage, are also under investigation as potential risk factors for decreased bone mineral density (BMD) frequently observed in SLE patients [10,11].

Studies of the pathogenesis of accelerated atherosclerosis in SLE patients are confounded by complex SLE-related factors. As a result, murine models have been developed to simultaneously express both atherosclerosis and lupus-like manifestations on either normal chow or high fat diet [7,12,13]. Apolipoprotein E-deficient (apoE^-/-^) C57BL/6 (B6) mice are established models of atherosclerosis that develop advanced atherosclerotic lesions when kept on a high fat diet [14]. Mice that are homozygous for *lpr *(lymphoproliferation or Fas^lpr/lpr^) or *gld *(generalized lymphoproliferative disease or FasL^gld/gld^) develop lymphadenopathy and present symptoms of lupus-like autoimmunity [7,15]. These symptoms include IgG autoantibodies commonly elevated in SLE patients, which result from mutations in Fas, a cell-surface protein that mediates apoptosis, or its ligand, FasL. We previously established the apoE^-/- ^and Fas^lpr/lpr ^(Fas^-/-^) double knockout B6 mouse as a model of accelerated atherosclerosis in lupus [16]. Compared to single knockout parental strains, double knockouts, fed a normal chow diet, simultaneously exhibit advanced accelerated atherosclerosis, glomerulonephritis, osteopenia, and lupus-like autoimmunity starting at five months of age [16].

Statins, 3-hydroxy-3-methylglutaryl-coenzyme A (HMG-CoA) reductase inhibitors involved in cholesterol biosynthesis, are widely used as lipid-lowering agents in the treatment of hypercholesterolemia and have been reported to possess anti-inflammatory and immunomodulatory properties [17]. Interestingly, statin treatments are not lipid-modulating in rodents as is commonly observed in humans, allowing focus to remain on potential anti-inflammatory and immunomodulatory effects [18]. Independent of cholesterol-lowering effects, daily injections of simvastatin (intraperitoneally (i.p.) 0.125 mg/kg/day) in young *gld*.apoE^-/-^ B6 mice maintained on a high-fat diet for 12 weeks prevented the development of both atherosclerosis and lupus-like disease via a shift from Th1 to Th2 phenotype [7,19]. Similarly, mono-therapy of oral pravastatin inhibited atherogenesis and plaque rupture in apoE^-/-^ B6 mice at high doses (≥ 40 mg/kg in drinking water) [20,21] and at low doses (≤ 5 mg/kg) in combination with additional therapy [20,22,23].

Apolipoprotein A-1 (apoA-1), a major component of high-density lipoproteins (HDL), plays an important role in the anti-inflammatory effects of HDL and mediates protection against atherosclerosis in animal models [24-26]. The apoA-1 mimetic peptide 4F, synthesized from either D (D-4F) or L (L-4F) amino acids, promotes the ability of HDL to protect low-density lipoprotein (LDL) from oxidation in animal models of atherosclerosis [27]. Oral administration of D-4F converts HDL from proinflammatory to anti-inflammatory, improves HDL-mediated cholesterol efflux, reverses transport of cholesterol from macrophages, and reduces aortic lesions in apoE^-/- ^mice without affecting plasma cholesterol levels [23,28,29].

Synergistic effects of suboptimal dosages of pravastatin and D-4F have been shown to inhibit atherogenesis in young apoE^-/- ^mice and to reduce lesion progression of established plaques in older mice where mono-therapies of pravastatin or D-4F alone were unsuccessful [23]. Here, low dose L-4F was administered i.p. (due to its rapid degradation by gut proteases when administered orally) [27]. Using a combination treatment of L-4F and pravastatin, we assessed the therapeutic effects of both drug types in the apoE^-/-^Fas^-/- ^murine model of accelerated atherosclerosis in lupus and identified potential biomarkers of disease activity for possible future applications in the treatment and monitoring of atherosclerosis in SLE.

## Materials and methods

### L-4F and pravastatin

L-4F was synthesized similar to the methods previously described [30,31]. Pravastatin sodium was purchased from LKT Laboratories, Inc. (St. Paul, MN, USA).

### Mice and experimental protocol

**Figure 1:**
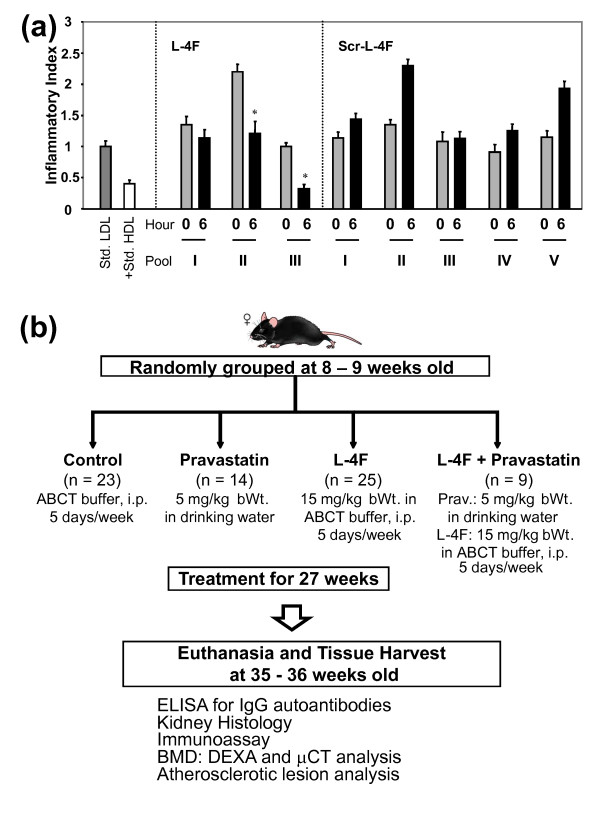
**Preliminary studies and experimental protocol**. **(a) **Preliminary studies to determine the use of L-4F as a potential treatment in apoE^-/-^Fas^-/- ^mice showed that HDL taken six hours after injection of L-4F was more successful in reducing LDL-induced monocyte chemotactic activity in cultures of human aortic endothelial cells compared to scrambled L-4F (Scr-L-4F). The value for No Addition (no LDL or HDL added to endothelial cultures) was subtracted from all values, the value for Std. LDL was taken as 1.0 and inflammatory index for LDL + HDL was calculated. Each pool represents HDL fractions from three to four mice. **(b) **Thirty-six week experimental protocol. *P ≤ 0.05.

ApoE^-/-^Fas^-/- ^B6 mice were originally produced by breeding apoE^-/- ^and Fas^-/- ^single knockout mice purchased from the Jackson Laboratories (Bar Harbor, ME, USA) and then further maintained in a colony [16]. At eight to nine weeks of age, female apoE^-/-^Fas^-/- ^mice were randomly grouped to receive one of four different treatment regimens: 1) pravastatin (5 mg/kg body weight in drinking water, n = 14), 2) L-4F (15 mg/kg in 50 mM ammonium bicarbonate buffer, pH 7.0, containing 0.1 mg/ml Tween-20 (ABCT) i.p., five days/week, n = 25), 3) L-4F plus pravastatin (administered as described for groups 1 and 2, n = 9), and 4) vehicle control (ABCT i.p., five days/week, n = 23) (Figure 1b). After 27 weeks, mice were fasted overnight and euthanized. At time of death, blood samples were collected via cardiac puncture. The mice were profused using phosphate buffered saline (PBS) (9.5 mM phosphate, pH 7.4, 2.7 mM KCl and 137 mM NaCl) prior to harvest of the spleen, lymph nodes, and kidneys (Figure 1b). All mice were treated in conformity with Public Health Service Policy. The mice were fed normal chow diet and maintained in a temperature-controlled room with a 12-hour light/dark cycle according to the approved protocol by the University of California, Los Angeles Animal Research Committee.

### Autoantibody analysis using enzyme-linked immunosorbant assay (ELISA)

Serum and plasma samples were collected from each mouse at euthanasia. An ELISA kit was used to test relative levels of total IgG antibodies. Serum samples were diluted 1:200 to measure relative levels of IgG anti-dsDNA using a streptavidin-biotin method of ELISA, and an IgG anti-cardiolipin ELISA was used to measure levels of IgG antibodies to oxidized phospholipids (oxPLs) -1-palmitoyl-2(5-oxovaleroyl)-*sn*-glycero-3-phosphorylcholine (POVPC) and 1-palmitoyl-2-glutaroyl-*sn*-glycero-3-phosphorylcholine (PGPC) - as previously described [16]. A standard curve was generated using serially diluted pooled sera from mice with known high concentrations of the desired antibody. Samples were measured using a goat anti-mouse IgG Fc antibody conjugated with either alkaline phosphatase enzyme or horseradish peroxidase (Bethyl Laboratories, Inc.; Montgomery, TX, USA).

### Kidney histology

Following euthanasia, one kidney from each mouse was fixed in 10% formalin. The samples were embedded in paraffin, sectioned at 3 μm, and stained using either periodic acid-Schiff (PAS) or hematoxylin and eosin (H&E). Stained sections were photographed electronically with a microscope fitted with a digital camera (Nikon Eclipse 600, Melville, NY, USA), assigned anonymous identification numbers, and analyzed using computer-assisted imaging software (Image ProPlus; Media Cybernetics, Bethesda, MD, USA) by a blinded observer. Twenty-five to thirty glomeruli for each sample were observed in representative fields on duplicate slides and were measured to calculate the mean glomerular tuft size for each mouse.

### Proteinuria measurement

Morning urine was regularly collected from each mouse throughout the duration of the treatment protocol. Albustix strips (Bayer; Elkhart, IN, USA) were used to estimate proteinuria levels from fresh urine samples. Levels of proteinuria were expressed as follows: 0 = none, 1 = trace, 2 = approximately 30 mg/dl, 3 = approximately 100 mg/dl, 4 = approximately 300 mg/dl, and 5 = >2,000 mg/dl.

### BMD analysis and three-dimensional microtomography

Following euthanasia, female mice were subsequently scanned using dual-energy X-ray absorptiometry (DEXA) with a Lunar PIXImus2 Densitometer (GE Medical Systems; Madison, WI, USA). BMD was measured for the whole skeleton excluding the skull, the lumbar spine (L2 to L6), and the femurs. Femoral BMD was calculated by averaging the BMD measurements for both femurs; in cases in which the left femur was used for bone marrow RNA extraction, femoral BMD was based on the BMD of the right femur alone.

L5 vertebrae were extracted from a random sample of mice and fixed in formalin. The vertebrae were packed in 1× PBS for evaluation using three-dimensional microtomography (μCT) (μCT 40, Scanco Medical; Bassordorf, Switzerland) in 12 μm slices at a threshold of 275 nm. Bone volume density, trabecular number, connectivity density, trabecular thickness, and trabecular separation were measured.

### Atherosclerotic lesions and immunohistochemistry

The basal portion of the heart and the proximal aorta were harvested to assess atherosclerotic manifestations, embedded in Tissue-Tec OCT medium, frozen in liquid nitrogen, and stored at -80°C. Tissue from the aortic root was selected for evaluation since most studies involving mouse models of atherosclerosis use it as reference tissue for plaque evaluation. Serial 10 μm thick cryosections were stained with Oil Red O and hematoxylin, counterstained with fast green, and analyzed via light microscope for atheromatous lesions [16].

Serial 10 μm thick cryosections of aortic root were individually immunohistochemically stained for either 1) macrophages (rat anti-mouse CD68; Vector Labs, Burlingame, CA, USA), 2) α-actin (alkaline phosphatase-conjugated monoclonal anti-α-smooth muscle actin; Sigma) [32], 3) T-cells (rat anti-mouse CD4; Vector Labs), or 4) VCAM-1 (rat anti-mouse VCAM-1; AbD Serotec; Raleigh, NC, USA). Slides were treated as previously described by Roque et al. using a biotinylated anti-rat IgG secondary antibody and Avidin/Biotinylated Enzyme Complexes (ABC Elite; Vector Labs) and visualized using VECTOR Red (P-nitrophenyl phosphate; VECTOR Red substrate kit; Vector Labs) [32]. Negative controls were prepared by omission of the primary antibody.

The slides were analyzed using similar methodology listed under Kidney histology. Images were taken of three to six samples from duplicate slides, which were analyzed by a blinded observer to calculate a mean stained area per lesion area for each mouse. Additional slides were stained for various tissue components (elastic fibers, ground substance, muscle, collagen, and fibrinoid and fibrin) using a Movat pentachrome stain.

### Plasma lipid profiles and monocyte chemotaxis assay

Plasma samples collected during euthanasia were analyzed for lipid levels (triglycerides, total cholesterol, HDL cholesterol, non-HDL cholesterol, unesterified cholesterol, and free fatty acids) using enzymatic colorimetric assays as previously described [33]. Mouse HDL was isolated from pooled plasma samples before and six hours after injection of L-4F or scrambled L-4F (that is, identical amino acids as contained in L-4F but arranged in a random sequence that markedly reduces lipid binding) using fast-protein liquid chromatography (FPLC) fractionation [34]. In order to assess the anti-inflammatory properties of L-4F, 10 mice from both the control group and the L-4F-treated group were randomly selected, totaling 20 mice, and combined to form three pools (with three to four mice per pool) for each group. Mouse LDL was isolated by FPLC from pooled plasma samples from both groups and tested for its ability to induce monocyte chemotactic activity in cultures of human aortic endothelial cells as previously described [34]. Plasma samples were pooled for this assay in order to isolate sufficient concentrations of LDL and HDL particles; sample volumes obtained from individual mice did not provide adequate lipoprotein levels to determine monocyte chemotactic activity.

### Chemokine analysis and flow cytometry

Luminex-based beadarray (RodentMap version 1.6; Rules Based Medicine, Inc., Austin, TX, USA) was used to simultaneously assess for 69 different antigens in plasma samples from 8 to 16 randomly selected mice per group. Fifteen of the 69 assays were not present at detectable levels (calbindin, EGF, endothelin-1, FGF-9, GM-CSF, GST-α, GST-μ, INF-γ, IL-11, IL-12p70, IL-17, IL-2, IL-3, IL-4, and NGAL) (See Supplemental table S1 in Additional file 1 for a complete list of chemokines/cytokines included in the Luminex assay).

Fluorescence-activated cell sorting (FACS) analysis was performed on spleen samples from the four different treatment groups to identify potential changes in immune cell subsets. Multi-color flow cytometry analysis was used to characterize populations of B cells (CD19, T1, T2, FO, MZ, and plasma cells), T cells (CD4 and CD8), and NK, CD11c, and CD11b cells. After standard Fc blocking, the fluorochrome-conjugated anti-mouse antibodies that were used for staining included FITC-, PE-, PerCP-, and APC-conjugated antibodies to CD19 (MB19-1), IgM (II/41), IgD (11-26c [11-26]), CD21 (eBio8D9 (8D9)), CD23 (B3B4), B220 (RA3-6B2), CD93 (AA4.1), CD62L (MEL-14), CD4 (GK1.5), CD8 (H35-17.2), NK1.1 (PK136), CD11c (N418), Ly6C (HK1.4), CD11b (M1/70) (all eBioscience; San Diego, CA, USA), CD138 (281-2) (BD Biosciences; San Jose, CA, USA). Samples were acquired on a FACSCalibur flow cytometer (BD Biosciences) and analyzed using FloJo software (Tree Star, Ashland, OR, USA).

### Statistical analysis

Data was collected and analyzed using Excel (Microsoft Office) or Prism 3.0 (Graphpad, La Jolla, CA, USA). For comparisons between two groups, unpaired student's *t*-test was used if the variance was normally distributed; Mann-Whitney *U *test was used for comparisons with a variance that was not normally distributed. Comparisons made between three or more groups were performed using one-way ANOVA. All results are presented as mean ± SD; *P *< 0.05 was considered significant. For Luminex-based beadarray of 69 plasma antigens, Bonferroni correction was applied for detectable antigens (n = 54); as a result, *P *< 0.0009, as calculated by (*P *< 0.05)/(n = 54) = (*P *< 0.0009), was considered significant.

## Results

### Treatment protocol

In apoE^-/-^Fas^-/- ^B6 mice that develop accelerated atherosclerosis and autoimmunity, we used a dose of Apo-A1 mimetic peptide twice as much as previously used in apoE^-/- ^B6 mice [23,35]. To determine an effective form of L-4F peptide, two groups of eight-week old double knockout mice (n = 10 per group) were fasted overnight, bled the following morning (0 h), injected with either 15 mg/kg i.p. L-4F or scrambled L-4F peptide, and harvested for blood samples six hours later. Compared to 0 h time point, two out of three blood sample pools from the L-4F group (three to four mice per pool), but none of the five sample pools from the scrambled L-4F group (two mice per pool), showed significant reduction in monocyte chemotactic activity after six hours (Figure 1a). These data suggest that 15 mg/kg of i.p. L-4F could improve HDL anti-inflammatory activity in young apoE^-/-^Fas^-/- ^mice. Suboptimal dosage of pravastatin was determined as previously described by Navab et al. [23]. This suboptimal dose was administered in order to prevent masking potential additive synergistic effects contributed by L-4F.

### Suppression of lupus-like autoimmunity with L-4F

After 26 to 27 weeks of treatment with 1) pravastatin, 2) L-4F, 3) L-4F plus pravastatin, or 4) vehicle control, mice treated with L-4F or L-4F plus pravastatin showed improved lupus-like autoimmune manifestations compared to vehicle controls.

**Figure 2:**
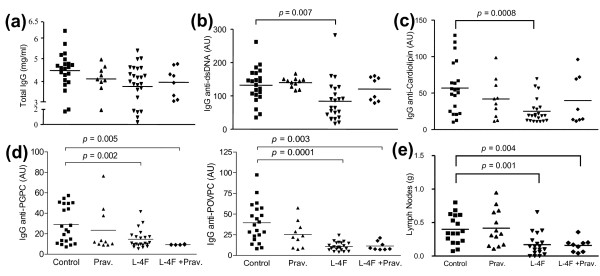
**Decreased auto-immune symptoms presented in mice treated with L-4F or combination treatment**. ELISA assays on serum from randomly selected female apoE^-/-^Fas^-/- ^mice at Week 35 or 36 showed: **(a) **comparable total serum IgG antibody levels among the different groups, suggesting an absence of general immune suppression, significantly reduced levels of **(b) **IgG anti-dsDNA and **(c) **IgG anti-cardiolipin in L-4F-treated mice, and **(d) **significantly lower IgG anti-PGPC and IgG anti-POVPC in mice treated with L-4F in the absence/presence of pravastatin. Pravastatin alone did not have any significant effect on IgG anti-dsDNA or IgG anti-oxPL levels. **(e) **In addition, lymph nodes from L-4F or L-4F plus pravastatin-treated mice were significantly smaller compared to control mice. Each symbol represents an individual mouse and the horizontal line represents the mean value. *P*-values < 0.05 were considered significant. AU, arbitrary units.

There was no significant difference in total IgG levels among the four groups, suggestive of no general immune suppression (Figure 2a). Serum levels of IgG anti-dsDNA antibodies and IgG anti-cardiolipin were significantly reduced in mice treated with L-4F (Figure 2b, c). Similarly, mice treated with L-4F, with or without pravastatin, had significantly lower serum levels of IgG autoantibodies to oxPLs--PGPC and POVPC compared to vehicle controls (Figure 2d). Although it appeared that pravastatin caused a mild canceling effect in combination treated mice, there was no significant difference in circulating levels of IgG anti-dsDNA and IgG anti-cardiolipin found between L-4F-treated mice and combination treatment mice.

Significantly smaller lymph nodes were present in both L-4F and L-4F plus pravastatin-treated mice compared to vehicle controls (0.17 ± 0.17 g and 0.16 ± 0.10 g vs. 0.40 ± 0.22 g; *P *= 0.001 and 0.004, respectively) (Figure 2e). However, upon comparison between treatment groups and vehicle controls, there was no significant difference in spleen size or splenocyte populations of B-cells, CD4+, CD8+ T-cells, NK, CD11c, CD11b cells as determined by multi-color flow analysis (data not shown).

**Figure 3:**
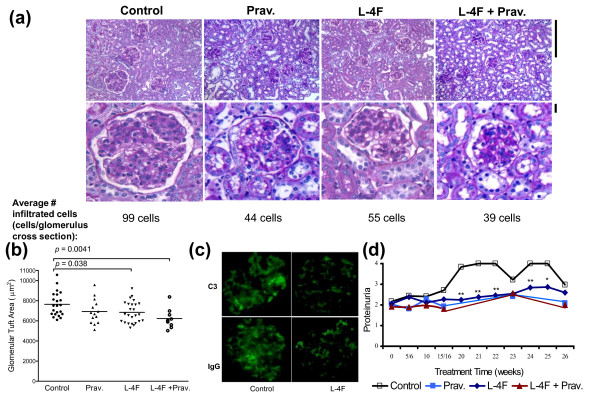
**Improved renal lesions in female apoE^-/-^Fas^-/- ^mice treated with L-4F or L-4F plus pravastatin**. **(a) **Glomeruli of female mice treated with L-4F or L-4F plus pravastatin had smaller glomerular tufts compared to vehicle controls as seen in representative fields of renal cortex from each group (top panel; PAS stain; magnification ×400) and enlarged images from the corresponding field (bottom panel). Bars = 25 μm. In addition, the average number of infiltrated glomerular cells reflected this trend. **(b) **Quantification of glomerular tuft showed mice treated with L-4F or L-4F plus pravastatin had significantly decreased glomerular tuft area compared to vehicle controls (6,845 ± 1,060 and 6,226 ± 1,007 μm^2 ^vs. 7,645 ± 1,200 μm^2^, respectively). **(c) **Immunofluorescence staining showed decreased deposition of IgG and C3 within kidneys of L-4F-treated mice compared to vehicle controls. **(d) **Starting Week 20 of treatment and through euthanasia, L-4F-treated mice had significantly lower levels of proteinuria compared to vehicle controls. *P ≤ 0.05; **P ≤ 0.01.

Kidney disease was followed non-invasively via analysis of proteinuria levels during the course of treatment. L-4F treatment was associated with lower proteinuria levels than in vehicle controls starting at Week 20 of the treatment protocol (Figure 3d). Upon histological analysis, the controls also had increased glomerular cell infiltration, analogous to diffuse proliferative glomerulonephritis (DPGN) in SLE patients (Figure 3a) [16]. L-4F or L-4F plus pravastatin-treated mice had decreased glomerular tuft size compared to vehicle controls (6,846 ± 1,062 μm^2 ^and 6,227 ± 1,007 μm^2 ^vs. 7,645 ± 1,201 μm^2^; *P *= 0.02 and 0.004, respectively), and combination treatment proved to be the most successful in preventing enlarged glomerular tufts (Figure 3a, b). Finally, immunofluorescence staining showed decreased amounts of IgG and C3 deposition in kidney sections of L-4F-treated mice compared to control mice (Figure 3c).

### Prevention of BMD loss and trabecular bone decay with L-4F treatment

**Figure 4:**
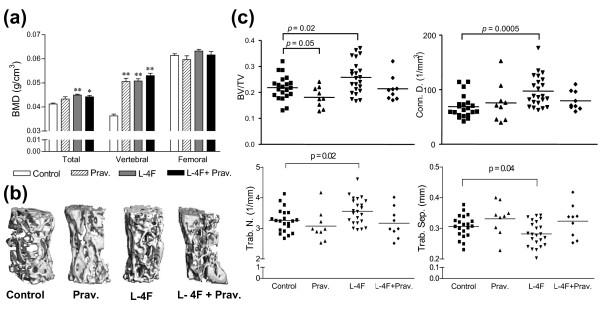
**Increased bone mineral density and decreased osteopenia in L-4F and L-4F plus pravastatin treated mice**. **(a) **Total and vertebral BMD (L2-L6), measured using DEXA, was increased in 35 to 36 week-old female apoE^-/-^Fas^-/- ^mice when treated with pravastatin, L-4F, or in combination when compared to vehicle controls (total: 0.041 ± 0.002 vs. 0.043 ± 0.002 and 0.044 ± 0.002 and 0.044 ± 0.002 g/cm^3^, respectively and vertebral: 0.036 ± 0.004 vs. 0.051 ± 0.005 and 0.051 ± 0.005 and 0.053 ± 0.003 g/cm^3^, respectively). **(b) **μCT images of L5 lumbar vertebrae from female mice at 35 to 36 weeks of age. Mice treated with L-4F showed significant improvement in trabecular bone content. **(c) **Three-dimensional morphometric evaluation of L5 vertebrae. Mice treated with L-4F had significantly increased bone volume density (BV/TV), connectivity density (Conn. D.), and trabecular number (Trab. N.) and significantly lower trabecular separation (Trab. Sep.) when compared to controls. *P ≤ 0.01; **P ≤ 1E-07.

Compared to vehicle controls, total skeletal BMD (excluding the skull) and lumbar BMD, measured using DEXA, were significantly higher in female mice treated with pravastatin, L-4F, or L-4F plus pravastatin (total: 0.041 ± 0.002 vs. 0.043 ± 0.002 and 0.044 ± 0.002 and 0.044 ± 0.002 g/cm^3^, respectively and vertebral: 0.036 ± 0.004 vs. 0.051 ± 0.005 and 0.051 ± 0.005 and 0.053 ± 0.003 g/cm^3^, respectively), with no significant difference between the pravastatin, L-4F, and L-4F plus pravastatin-treated groups (Figure 4a). Additionally, there were no apparent treatment-dependent effects on femoral BMD. Concurrent μCT analysis showed that mice treated with L-4F had significantly higher bone volume density (*P *= 0.023), trabecular number (*P *= 0.019), and connectivity density (*P *= 0.00054) and significantly lower trabecular separation compared to vehicle controls (*P *= 0.04) (Figure 4b, c). In contrast, treatment with pravastatin alone was associated with a borderline reduction in bone volume density, and treatment with L-4F plus pravastatin did not show significant improvements in any of these trabecular characteristics.

### Enlarged atheromas in L-4F-treated mice

**Figure 5:**
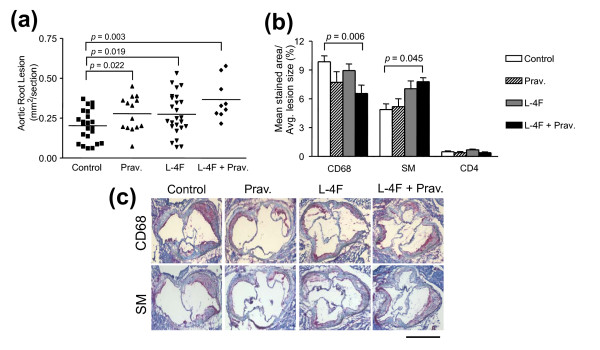
**Evaluation of atherosclerotic manifestations**. **(a) **Larger aortic lesions were seen in mice treated with pravastatin or L-4F or L-4F plus pravastatin when compared to vehicle controls (0.28 ± 0.11 and 0.27 ± 0.13 and 0.37 ± 0.13 μm^2 ^vs. 0.19 ± 0.10 μm^2^, respectively). **(b) **Aortic lesions from L-4F plus pravastatin treated mice had significantly decreased macrophage infiltration when compared to vehicle controls (6.2 ± 1.2 vs. 9.8 ± 0.8%, respectively; *P *= 0.006). In addition, increased smooth muscle content in combination treatment mice compared to vehicle controls (7.8 ± 0.5% vs. 4.9 ± 2.3%, respectively; *P *= 0.04) suggests possible plaque remodeling. CD4^+ ^T cell levels appeared unaltered by treatment. **(c) **Ten micrometer aortic root sections from female mice were stained for macrophage infiltration (CD68; rat anti-mouse CD68) and smooth muscle cells (SM, rat anti-mouse α-smooth muscle actin). Bar = 1 mm.

Following 27 weeks of treatment then euthanasia, the basal portion of the heart and the proximal aorta showed enlarged aortic lesions in mice treated with pravastatin, L-4F, or L-4F plus pravastatin compared to controls (Figure 5a). Analysis of local plaque environment composition at the aortic root demonstrated significantly decreased CD68+ macrophage infiltration, when comparing the average total stained area per mean lesion area, in L-4F plus pravastatin-treated mice compared to age-matched vehicle controls (6.2 ± 1.2% vs. 9.8 ± 0.8%; *P *= 0.002) (Figure 5b, c). L-4F plus pravastatin-treated mice also showed increased α-actin smooth muscle content in aortic lesions compared to controls (7.8 ± 0.5% vs. 4.9 ± 2.3%; *P *= 0.04) (Figure 5b, c). Mice treated with pravastatin or L-4F alone did not show any significant improvements in aortic lesion cellular composition compared to control mice. Analysis of Movat, CD4+ T-cell, or VCAM-1 stained lesions did not show any significant differences in atheroma composition of elastic fibers, ground substance, muscle, collagen, fibrinoid and fibrin (See Supplemental figure S1 in Additional file 2 for Movat staining of aortic root atheromas), CD4+ T-cells or VCAM-1 distribution between any of the treatment groups and the control group (data not shown).

### Plasma lipid profiles and decreased proinflammatory lipoprotein activity with L-4F treatment

**Figure 6:**
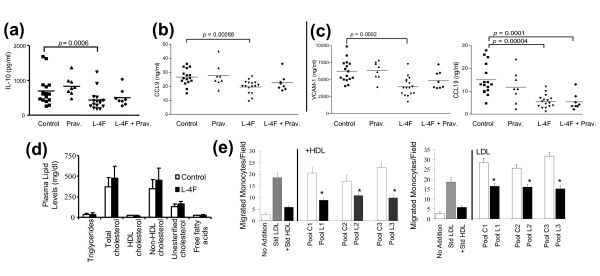
**Unaffected lipid profiles with modified plasma antigen levels and monocyte chemotactic activity in representative mice**. Luminex-based bead array was performed for plasma chemokines and cytokines, including: **(a) **IL-10 (interleukin-10; also known as human cytokine synthesis inhibitory factor, CSIF), a cytokine secreted in response to tissue damage, presented lower levels in L-4F-treated mice--consistent with increased tissue damage in control mice. **(b) **Plasma levels of CCL9 (also known as MIP-1γ), a chemoattractant that contributes to monocyte infiltration in renal disease, were significantly less in mice treated with L-4F. **(c) **Indicators of atherosclerosis severity: CCL19 (also known as MIP-3-β) and VCAM-1. CCL19 recruits T-cells and dendritic cells to target organs and promotes inflammatory responses and was significantly decreased in mice treated with L-4F or combination treatment. Similar trends were seen with VCAM-1, an endothelial adhesion molecule involved in atherosclerotic plaque formation and progression of glomerulonephritis. After Bonferoni correction, *P*-values less than 0.0009 for plasma markers were considered significant. **(d) **Plasma lipid levels, including total cholesterol, HDL cholesterol, and non-HDL cholesterol, were unaffected in all of the treatment groups compared to vehicle controls. **(e) **However, L-4F (L) significantly rendered mouse HDL anti-inflammatory and LDL less inflammatory compared to control (C) as determined in cultures of human aortic endothelial cells (n = 10 mice per treatment group, three to four mice per pool). *P ≤ 0.05.

Plasma lipid profiles for apoE^-/-^Fas^-/- ^L-4F-treated mice and vehicle control mice did not show any significant differences in triglyceride, total cholesterol, HDL cholesterol, non-HDL cholesterol, unesterified cholesterol, and free fatty acid levels (Figure 6d). L-4F improved the anti-inflammatory function of plasma HDL and decreased the proinflammatory effects of LDL from mice injected with L-4F as determined in cultures of human aortic endothelial cells compared to LDL from vehicle control mice (Figure 6e).

### Circulating plasma chemokines and cytokine levels remained mostly unaffected by L-4F treatment

To explore potential biomarkers associated with treatment response, plasma from female apoE^-/-^Fas^-/- ^mice was analyzed for 69 chemokines and cytokines using Luminex-based beadarray. L-4F treatment resulted in a trend toward decreased levels of tissue damage and inflammation indicators, including CRP (C-reactive protein), fibrinogen, TNF-α (tumor necrosis factor-alpha), and CCL12 (monocyte chemotactic protein 5 (MCP-5)), when compared to control mice (data not shown).

After Bonferroni correction for multiple testing (54 detectable antigens), plasma levels of IL-10 (interleukin-10) - a cytokine secreted in response to damaged tissue through growth and differentiation of NK and B cells and CCL9 (macrophage inflammatory protein-1γ (MIP-1γ)) - a chemoattractant for monocytes, neutrophils, and macrophages that contributes to monocyte infiltration in renal disease, were significantly lower in L-4F-treated mice (Figure 6a, b). Decreased levels of CCL19 -- a homeostatic interferon-regulated chemokine that binds to CCR7 and plays a role in recruiting T-cells and dendritic cells to target organs, promoting inflammatory responses, and unstable plaque formation in atherosclerosis [36] -- were present in mice treated with L-4F compared to control mice (Figure 6c). Similarly, the endothelial receptor VCAM-1, commonly associated with the recruitment of monocytes and lymphocytes during atherosclerotic plaque formation [37], was significantly decreased in plasma of mice treated with L-4F, as compared to control mice (Figure 6c). Pravastatin monotherapy alone did not significantly affect any of the levels of these circulating chemokines and cytokines.

## Discussion

Treatment with L-4F, in the absence or presence of pravastatin, effectively reduced manifestations of lupus-like autoantibody production, glomerulonephritis, and osteopenia in our apoE^-/-^Fas^-/- ^B6 murine model of accelerated atherosclerosis in SLE. Only mice treated with L-4F, with or without pravastatin, had significantly reduced glomerular tuft size, IgG anti-PGPC and IgG anti-POVPC antibodies, lower plasma proinflammatory cytokine/chemokine levels, and increased total and vertebral BMD compared to vehicle controls. Furthermore, mice treated with L-4F alone also had significantly lower levels of IgG anti-dsDNA and IgG anti-cardiolipin autoantibodies. Although larger aortic lesions were consistently present in all the treatment groups, lesion characteristics of the combination treatment group indicate decreased macrophage infiltration and inflammation, potentially suggestive of plaque remodeling. Despite the reported success of the immunomodulatory effects of statins in mouse models, no increased effects were appreciated in mice treated with the combination treatment compared to those receiving L-4F alone. To our knowledge, our L-4F treatment regimen has not been previously used in murine models of atherosclerosis in SLE.

Statins in SLE patients and murine models have shown varying degrees of success in recent trials [7,38-40]. Pravastatin was successful in reducing total cholesterol and LDL at both 10 mg/day and 40 mg/day doses, but failed to exhibit anti-inflammatory properties in rheumatoid arthritis patients [38]. Conversely, atorvastatin showed positive results in the prevention of endothelial-dependant vasodilation and reduction in disease activity in SLE patients at 20 mg/day in a controlled trial, but failed as a mono-therapy in a NZB/NZW murine lupus model to control anti-dsDNA antibodies, proteinuria, and kidney disease [39,41]. Nachtigal et al. mentions that compared to human studies, higher doses of statins are normally required in mouse models; this is potentially a result of the inactivation of HMG-CoA reductase inhibitors by P450 enzyme induction and the elevation of HMG-CoA reductase levels [42-44]. These studies suggest that the efficacy of statins as treatment for systemic inflammation, characteristic of SLE, is dependent on the study protocol, dosage, and/or inclusion/exclusion criteria for study participation. In our attempt to achieve synergistic effects between our statin regimen and our administered novel peptide, our suboptimal dose of pravastatin alone did not significantly control the progression of either renal deterioration, production of IgG autoantibodies to dsDNA or oxPLs, or formation of atherosclerotic lesions in our model.

Since statin regimens have had such varied results among different studies, we added an apolipoprotein mimetic peptide to potentially contribute pleiotropic effects as seen in other murine models of atherosclerosis [23]. Recent studies have shown the effectiveness of piHDL as a predictor of subclinical atherosclerosis in SLE patients [45,46]. Since L-4F effectively reduced the proinflammatory effects of LDL in preliminary studies (Figure 1a), we believed L-4F could potentially be utilized to target inflammatory lipids and as a result, limit the progression of inflammation, including atherosclerotic manifestations, in our lupus model.

Renal involvement and glomerulonephritis are serious complications that can present in patients diagnosed with SLE. Elevated plasma levels of VCAM-1, which also plays a role in perpetuating atherosclerotic plaque formation, are associated with nephritis and increased disease activity in SLE patients [37]. Similarly, Yao et al. proposed a correlation between increased renal lesions, elevated levels of VCAM-1, and degree of symptom severity in patients with lupus nephritis [47]. In our study, lower circulating VCAM-1 levels were consistent with 11% and 19% smaller mean glomerular tuft areas seen in L-4F or combination treatment mice, respectively, compared to vehicle controls after 27 weeks of treatment.

L-4F treatment in the presence or absence of pravastatin also significantly prevented overall bone loss and additional osteopenic manifestations within the lumbar spine, as reflected in significantly higher total BMD and vertebral BMD in treatment mice, compared to vehicle controls. Feng et al. showed that five-month-old female apoE^-/-^Fas^-/- ^mice experienced a greater decrease in vertebral BMD than in femoral BMD by the time they reached nine months [16]; this could account for the minimal difference seen among the femoral BMD values of the different treatment groups. Okamatsu et al. previously demonstrated, in a series of neutralization studies, that RANKL, a stimulator of osteoclastogenesis, activation, and survival, triggers CCL9, which further stimulates osteoclast activation for bone resorption [48]. Mice receiving L-4F had significantly lower plasma levels of CCL9 than control mice, which correspond with improved trabecular bone characteristics observed in L-4F-treated mice compared to vehicle controls. Furthermore, Graham et al. demonstrated that the production of RANKL, by T lymphocytes could be induced by circulating oxPLs [49], indicating that osteopenic manifestations could be linked to atheroma formation as a result of elevated levels of circulating oxPLs.

OxPLs, such as POVPC and PGPC, are commonly found in oxidized LDL and aid in the development of fatty streaks, which may contribute to accelerated atherosclerosis in SLE [50]. Mice with L-4F or combination treatment showed significantly decreased levels of IgG autoantibodies to both POVPC and PGPC without significant alteration in plasma lipid levels (Figure 6d). In addition, L-4F successfully improved the anti-inflammatory function of HDL and reduced the proinflammatory nature of LDL, as determined in cultures of human aortic endothelial cells. Increased levels of circulating CCL19 has been correlated with unstable plaques in patients with CVD compared to patients with stable plaques [36]. Significantly decreased levels of circulating CCL19 and VCAM-1, both linked to plaque formation and instability, are consistent with possibly improved lesion characteristics in both L-4F and combination treatment mice.

Despite reduced inflammation, as indicated by lower levels of circulating plasma proinflammatory chemokines and reduced lipoprotein inflammatory activity in cultures of human aortic endothelial cells, all treatment groups presented enlarged aortic lesions compared to vehicle controls. In response, we investigated the composition of the local plaque environment at the aortic root to determine the relationship between size and stability of the atheromas in our model. In humans, advanced plaques, which are more vulnerable to rupture, are characterized by large populations of infiltrated macrophages, lower concentrations of smooth muscle cells, and lower collagen content with thinner fibers [51,52]. Aortic lesions from mice treated with L-4F plus pravastatin had 37% less mean macrophage area and 59% more mean smooth muscle cell area compared to vehicle controls (Figure 6b). Although these characteristics indicate improved plaque composition, tissue levels of VCAM-1 did not reflect the significantly decreased levels seen in circulation in L-4F-treated mice and showed minimal deviation across the four treatment groups. Similarly, collagen content of lesions from the different groups did not vary significantly. Despite this, the improved changes in atheroma cellular composition of both infiltrating macrophages and SMC content, in combination with circulating levels of IgG anti-oxPLs and atherogenic chemokines, possibly suggest improved stability and potential remodeling of atherosclerotic lesions in L-4F-treated mice compared to vehicle controls.

The histopathologic composition of the lesions indicated that oil red O and α-actin stained areas predominantly contributed to the increased lesion size in the three treatment groups compared to the control group. This may be the result of elevated neutral triglycerides and lipids (as reflected by oil red O staining) and increased smooth muscle (as reflected by α-actin staining). However, there was no apparent increase in volume of infiltrated CD68+ macrophages, collagen content, and t-cell concentration in any of the treatment groups compared to the control group that could have contributed to the increased lesion size. In a previous study, daily treatment with oral pravastatin (50 μg/mouse) and subcutaneously injected L-4F (200 μg/mouse) did not show improvement of aortic plaques in a small cohort of nine-month-old female ApoE^-/- ^B6 mice compared to baseline studies [53]. Similarly, daily suboptimal doses of oral atorvastatin (10 mg/kg) have been shown to mildly increase plaque size, albeit not significantly, in two-month-old female apoE^-/-^LDL^-/- ^mice when compared to controls [54].

Circulating proinflammatory cytokines and chemokines trended lower in mice receiving L-4F with or without pravastatin. The presence of circulating markers in the control mice, such as IL-10, a Th2 cytokine involved in B cell activity upregulation and linked to increased IgG anti-dsDNA autoantibodies [55], CCL19, and VCAM-1, indicate increased autoimmune response and increased risk for unstable atherosclerotic plaques due to their role in humoral immunity or monocyte recruitment to plaque sites [36,37,55,56]. Reduction of these circulating cytokines by L-4F or combination treatment may have contributed to limiting inflammation-induced glomerulonephritis by dampening autoimmune responses in our mice. Biomarkers such as these could potentially be developed into a chemokine score to monitor and assess disease activity in patients with SLE and atherosclerosis. Similarly, Bauer et al. proposed that monitoring CCL19 and other interferon-regulated chemokines would be beneficial for the assessment of current disease activity and prediction of future flares in patients with SLE [57].

## Conclusions

L-4F in the presence or absence of pravastatin reduced IgG anti-dsDNA, IgG anti-oxPLs, and IgG anti-cardiolipin antibody production and symptoms of glomerulonephritis and osteopenia in our apoE^-/-^Fas^-/- ^B6 murine lupus model of accelerated atherosclerosis. In addition, despite enlarged aortic atheromas present in all treatment groups, analysis of plaque composition is suggestive of potential remodeling. Atherosclerosis and its clinical manifestations are major contributors to morbidity and mortality in women with SLE. While traditional risk factors cannot fully predict the risk associated with the development of accelerated atherosclerosis in SLE, new mouse models, such as our apoE^-/-^Fas^-/- ^B6 model, that exhibit both autoimmune manifestations and advanced atherogenesis, may aid in the understanding of pathways that contribute to the onset and progression of systemic autoimmune diseases with cardiovascular involvement.

## Abbreviations

ABCT: 15 mg/kg in 50 mM ammonium bicarbonate buffer: pH 7.0: containing 0.1 mg/ml Tween-20; apoA-1: apolipoprotein A-1; apoE^-/-^: apolipoprotein E-deficient; B6: C57BL/6; BMD: bone mineral density; CVD: cardiovascular disease; DEXA: dual-energy X-ray absorptiometry; DPGN: diffuse proliferative glomerulonephritis; dsDNA: double stranded DNA; ELISA: enzyme-linked immunosorbant assay; FACS: fluorescence-activated cell sorting; Fas^-/-^: Fas^lpr/lpr^; FPLC: fast-protein liquid chromatography; HDL: high density lipoproteins; H & E: hematoxylin and eosin; HMG-CoA: 3-hydroxy-3-methylglutaryl-coenzyme A; IL: interleukin; i.p.: intraperitoneally; LDL: low density lipoproteins; MIP-1γ: macrophage inflammatory protein-1γ; oxPLs: oxidized phospholipids; PAS: periodic acid Schiff; PGPC: 1-palmitoyl-2-glutaroyl-*sn*-glycero-3-phosphorylcholine; PiHDL: proinflammatory high-density lipoproteins; POVPC: 1-palmitoyl-2(5-oxovaleroyl)-*sn*-glycero-3-phosphorylcholine; SLE/lupus: systemic lupus erythematosus; μCT: three-dimensional microtomography.

## Competing interests

Mohamad Navab and Alan M. Fogelman are principals in Bruin Pharma and Alan M. Fogelman is an officer in Bruin Pharma. The remaining authors have no competing interests.

## Authors' contributions

JW contributed to acquisition of data, performed data analysis and interpretation, and drafted the manuscript. ZL, CVD, YTL, KW, HL, LC, XW, NI, OR and HW contributed to acquisition and analysis of data. MN, ALC, AF and AL contributed to study conception and design and data interpretation. BT contributed to study conception and design, performed data analysis and interpretation, and helped draft the manuscript. All authors have read, revised and approved the final manuscript.

## Supplementary Material

Additional file 1 **Supplemental table S1**. Plasma samples from randomly selected mice were analyzed for 69 chemokines and cytokines using Luminex-base beadarray (RodentMap version 1.6; Rules Based Medicine, Inc.). *: Antigen not present at detectable levels. Click here for file

Additional file 2 **Supplemental figure S1**. Movat staining of lesions of the aortic root did not show any statistically significant differences in local plaque environment of the aortic root between any of the treatment groups and the vehicle controls. (Elastic fibers-black, ground substance-blue, muscle-red, collagen-yellow, and fibrinoid and fibrin-intense red). Bar = 1 mm. Click here for file
