# Supplementary material for: Treatment with apolipoprotein A-1 mimetic peptide reduces lupus-like manifestations in a murine lupus model of accelerated atherosclerosis
Source: Arthritis Res Ther. 2010 May 18;12(3):R93. doi: 10.1186/ar3020 (PMC2911877; doi:10.1186/ar3020)
Supplement: Additional file 1 — Supplemental table S1. Plasma samples from randomly selected mice were analyzed for 69 chemokines and cytokines using Luminex-base beadarray (RodentMap version 1.6; Rules Based Medicine, Inc.). *: Antigen not present at detectable levels. [file ar3020-S1.PDF]

**Luminex-based beadarray (RodentMap version 1.6; Rules Based Medicine, Inc.)**
Apo A1 (Apolipoprotein A1)
Beta-2 Microglobulin
*Calbindin
CD40
CD40 Ligand
Clusterin
CRP (C Reactive Protein)
Cystatin-C
*EGF (Epidermal Growth Factor)
*Endothelin-1
Eotaxin
Factor VII
*FGF-9 (Fibroblast Growth Factor-9)
FGF-basic (Fibroblast Growth Factor-basic)
Fibrinogen
GCP-2 (Granulocyte Chemotactic Protein-2)
*GM-CSF (Granulocyte Macrophage-Colony Stimulating Factor)
Growth Hormone
*GST-alpha (Glutathione S-Transferase alpha)
*GST-Mu
Haptoglobin
*IFN-gamma (Interferon-gamma)
IgA (Immunoglobulin A)
IL-10 (Interleukin-10)
*IL-11 (Interleukin-11)
*IL-12p70 (Interleukin-12p70)
*IL-17 (Interleukin-17)
IL-18 (Interleukin-18)
IL-1alpha (Interleukin-1alpha)
IL-1beta (Interleukin-1beta)
*IL-2 (Interleukin-2)
*IL-3 (Interleukin-3)
*IL-4 (Interleukin-4)
IL-5 (Interleukin-5)
IL-6 (Interleukin-6)
IL-7 (Interleukin-7)
Insulin
IP-10 (Inducible Protein-10)
KC/GROalpha (Melanoma Growth Stimulatory Activity Protein)
Leptin
LIF (Leukemia Inhibitory Factor)
Lymphotactin
MCP-1 (Monocyte Chemoattractant Protein-1)

MCP-3 (Monocyte Chemoattractant Protein-3)
MCP-5 (Monocyte Chemoattractant Protein-5)
M-CSF (Macrophage-Colony Stimulating Factor)
MDC (Macrophage-Derived Chemokine)
MIP-1alpha (Macrophage Inflammatory Protein-1alpha)
MIP-1beta (Macrophage Inflammatory Protein-1beta)
MIP-1gamma (Macrophage Inflammatory Protein-1gamma)
MIP-2 (Macrophage Inflammatory Protein-2)
MIP-3beta (Macrophage Inflammatory Protein-3beta)
MMP-9 (Matrix Metalloproteinase-9)
MPO (Myeloperoxidase)
Myoglobin
*NGAL (Lipocalin-2)
OSM (Oncostatin M)
Osteopontin
RANTES (Regulation Upon Activation, Normal T-Cell Expressed and Secreted)
SAP (Serum Amyloid P)
SCF (Stem Cell Factor)
SGOT (Serum Glutamic-Oxaloacetic Transaminase)
TIMP-1 (Tissue Inhibitor of Metalloproteinase Type-1)
Tissue Factor
TNF-alpha (Tumor Necrosis Factor-alpha)
TPO (Thrombopoietin)
VCAM-1 (Vascular Cell Adhesion Molecule-1)
VEGF (Vascular Endothelial Cell Growth Factor)
vWF (von Willebrand Factor)
