# Supplementary figures and images for: Treatment with apolipoprotein A-1 mimetic peptide reduces lupus-like manifestations in a murine lupus model of accelerated atherosclerosis
Source: Arthritis Res Ther. 2010 May 18;12(3):R93. doi: 10.1186/ar3020 (PMC2911877; doi:10.1186/ar3020)

## Slide 1
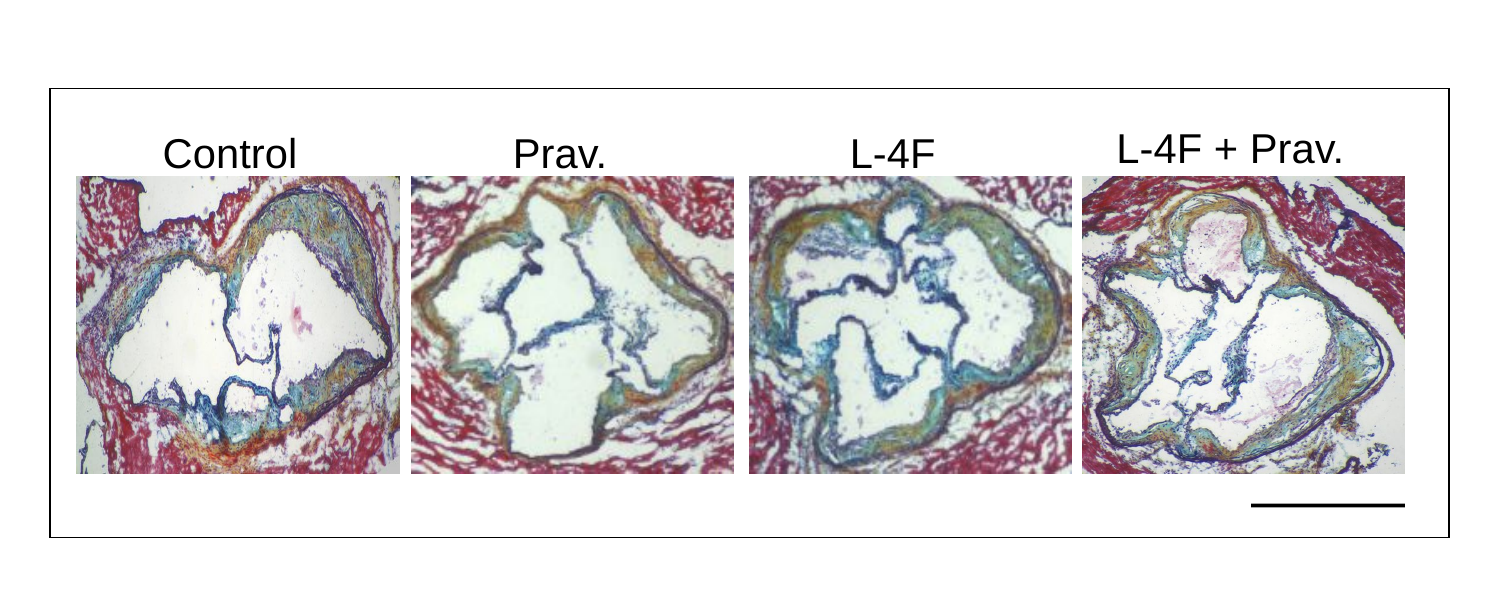

L-4F + Prav.
Prav.
Control
L-4F

Supplement: Additional file 2 — Supplemental figure S1. Movat staining of lesions of the aortic root did not show any statistically significant differences in local plaque environment of the aortic root between any of the treatment groups and the vehicle controls. (Elastic fibers-black, ground substance-blue, muscle-red, collagen-yellow, and fibrinoid and fibrin-intense red). Bar = 1 mm. [file ar3020-S2.PPT]
